# Supplementary material for: High Diversity of Human Non-Polio Enterovirus Serotypes Identified in Contaminated Water in Nigeria
Source: Viruses. 2021 Feb 5;13(2):249. doi: 10.3390/v13020249 (PMC7914538; doi:10.3390/v13020249)
Supplement: Supplementary file 1 [file viruses-13-00249-s001.zip › Figure S2.pdf]

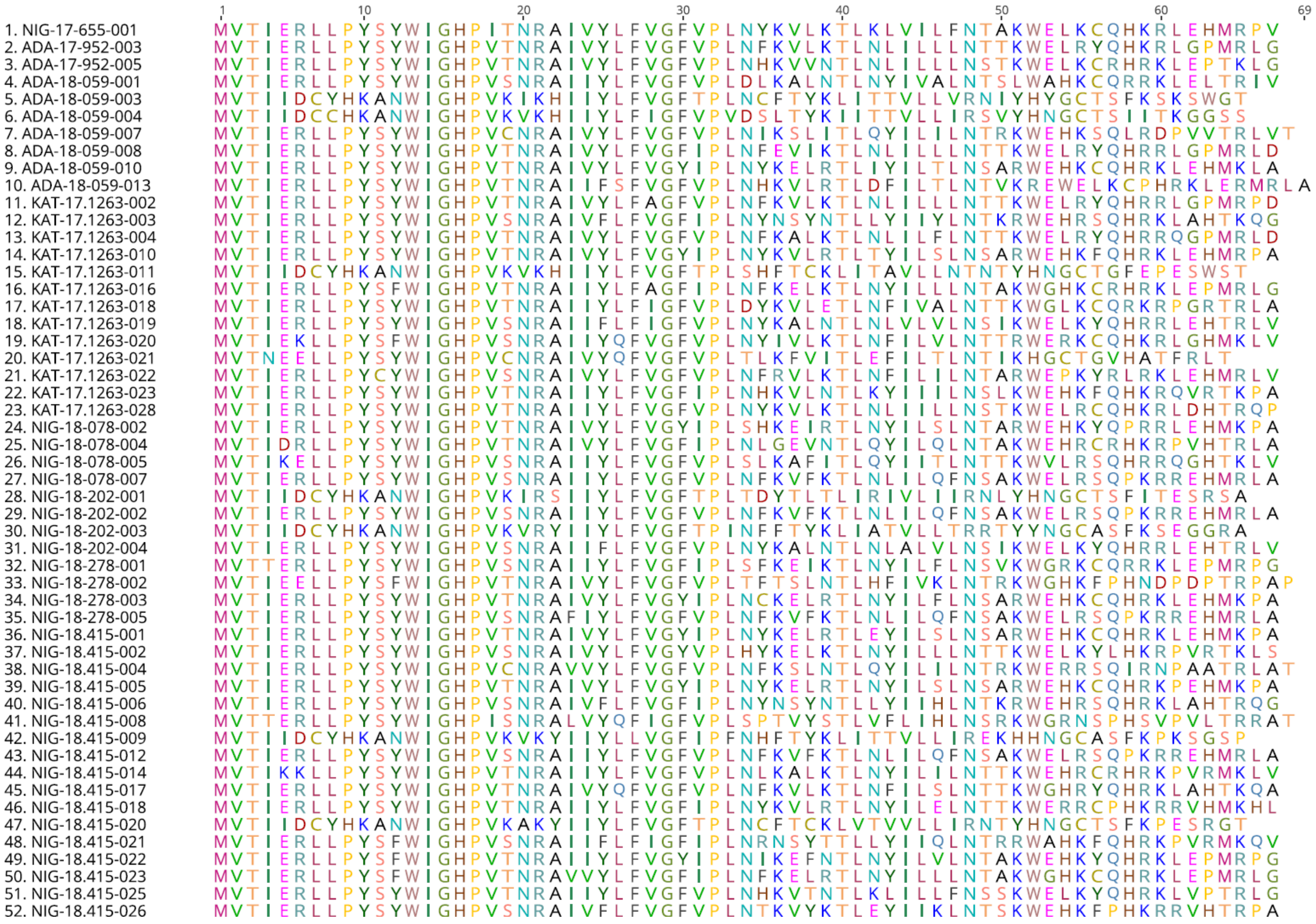

Supplementary Figure 2. uORF amino acid products from 52 EV sewage strains from Nigeria.
