# Supplementary material for: High Diversity of Human Non-Polio Enterovirus Serotypes Identified in Contaminated Water in Nigeria
Source: Viruses. 2021 Feb 5;13(2):249. doi: 10.3390/v13020249 (PMC7914538; doi:10.3390/v13020249)
Supplement: Supplementary file 1 [file viruses-13-00249-s001.zip › Table S1.pdf]

Table S1. Genetic properties of EV strains identified in sewage samples from Nigeria.

Sewage strain	Closest relative from NCBI sequence database				
	**Accession No.**	**% Identity**	**Year**	**Country**	**Serotype**
ADA-17-952-001	MN812198	93.3	2012	Ghana	EV-A119
ADA-17-952-002	JX417869	83.2	2008	Cameroon	CV-A24
ADA-17-952-003	KX580688	92.4	2014	Nigeria	E-14
ADA-17-952-004	MN812198	93.8	2012	Ghana	EV-A119
ADA-17-952-005	JX437649	91.7	2008	Chad	E-24
ADA-18-059-001	EF127247	84.1	Unknown	DRC	EV-B81
ADA-18-059-002	JX426639	88.2	2009	Cameroon	CV-A13
ADA-18-059-003	KF303095	87.9	2012	Nigeria	CV-A13
ADA-18-059-004	KF303095	91.1	2012	Nigeria	CV-A13
ADA-18-059-005	MH933853	83.5	2014	Cameroon	EV-C99
ADA-18-059-006	MH785183	87.9	2016	Nigeria	CV-A20
ADA-18-059-007	JQ364886	87.8	1992	China	CV-A6
ADA-18-059-008	KX580688	93.3	2014	Nigeria	E-14
ADA-18-059-009	KX765171	92.6	2015	Nigeria	EV-A119
ADA-18-059-010	KF541626	86.2	2008	Cameroon	E-15
ADA-18-059-011	KF413036	83.6	2007	India	CV-A12
ADA-18-059-012	MK086488	95.1	2014	France	CV-A19
ADA-18-059-013	KX580693	94.5	2014	Nigeria	EV-B97
KAT-17-1263-001	KC787153	93.3	2009	Cameroon	EV-A119
KAT-17-1263-002	KX580688	94.6	2014	Nigeria	E-14
KAT-17-1263-003	MK190418	96.2	2016	Nigeria	E-1
KAT-17-1263-004	KX580688	92.1	2014	Nigeria	E-14
KAT-17-1263-005	MK086469	86.8	2014	France	CV-A11
KAT-17-1263-006	MF686568	84.6	2015	Nigeria	CV-A13
KAT-17-1263-007	JX426647	84.3	2009	Nigeria	CV-A13
KAT-17-1263-008	JX426626	84.5	2008	Cameroon	CV-A13
KAT-17-1263-009	MF686568	85.1	2015	Nigeria	CV-A13
KAT-17-1263-010	KF541626	88.9	2008	Cameroon	E-15
KAT-17-1263-011	KY748288	88.6	2015	Nigeria	CV-A17

KAT-17-1263-012	JX417841	84.6	2009	Cameroon	CV-A13
KAT-17-1263-013	MK086476	88.5	2014	France	EV-C99
KAT-17-1263-014	JX426622	85.5	2008	Cameroon	CV-A13
KAT-17-1263-015	MG761738	96.7	2017	Nigeria	EV-A76
KAT-17-1263-016	KX580640	95.7	2014	Nigeria	E-7
KAT-17-1263-017	MN812189	87.4	2012	Ghana	EV-C99
KAT-17-1263-018	KY433761	92.8	2013	Niger	E-3
KAT-17-1263-019	KF698798	97.2	2012	Nigeria	E-19
KAT-17-1263-020	MH084296	89.2	2013	Senegal	EV-B106
KAT-17-1263-021	MG367600	97.4	2016	Denmark	EV-A71
KAT-17-1263-022	MH005794	89.3	2017	Ghana	E-6
KAT-17-1263-023	JX476202	88.2	2009	India	EV-B86
KAT-17-1263-024	KF413036	83.4	2007	India	CV-A12
KAT-17-1263-025	KX580675	90.8	2014	Nigeria	EV-B73
KAT-17-1263-026	MN812195	91.4	2012	Ghana	EV-B74
KAT-17-1263-027	MK190419	96.8	2017	Nigeria	EV-D111
KAT-17-1263-028	KX580667	96.6	2014	Nigeria	EV-B80
NIG-17-655-001	MT347976	96.5	2016	USA	E-25
NIG-17-655-002	MG761738	96.7	2017	Nigeria	EV-A76
NIG-18-078-001	KY798125cpz	98.7	2016	Nigeria	EV-A76
NIG-18-078-002	KF541626	86.5	2008	Cameroon	E-15
NIG-18-078-003	MG692407	86.3	2016	Ethiopia	CV-A14
NIG-18-078-004	JX417820	89.6	2008	Cameroon	EV-B87
NIG-18-078-005	AY843303	87.7	2000	Bangladesh	EV-B85
NIG-18-078-006	MN812195	92.3	2012	Ghana	EV-B74
NIG-18-078-007	MG845889	94.8	2017	USA	CV-B4
NIG-18-078-008	MK190419	95.2	2017	Nigeria	EV-D111
NIG-18-202-001	JX426637	87.5	2008	Cameroon	CV-A13
NIG-18-202-002	MG845889	96.3	2017	USA	CV-B4
NIG-18-202-003	MH785183	88.1	2016	Nigeria	CV-A20
NIG-18-202-004	KF698798	96.0	2012	Nigeria	E-19
NIG-18-202-005	MG252509	94.2	2015	Nigeria	CV-A19
NIG-18-278-001	MG761743	95.9	2017	Nigeria	E-9
NIG-18-278-002	KC787155	89.9	2009	Cameroon	CV-A4

NIG-18-278-003	KF541626	88.7	2008	Cameroon	E-15
NIG-18-278-004	KT285370	96.4	2014	Ivory Coast	EV-C116
NIG-18-278-005	MG845889	96.4	2017	USA	CV-B4
NIG-18-415-001	KF541626	88.3	2008	Cameroon	E-15
NIG-18-415-002	MG252518	92.6	2015	Nigeria	E-31
NIG-18-415-003	KT285370	96.6	2014	Ivory Coast	EV-C116
NIG-18-415-004	JQ364886	87.7	1992	China	CV-A6
NIG-18-415-005	KF541626	87.1	2008	Cameroon	E-15
NIG-18-415-006	MK190418	96.7	2016	Nigeria	E-1
NIG-18-415-007	MK086281	96.6	2014	France	CV-A5
NIG-18-415-008	MK836121	93.2	2018	USA	CV-A2
NIG-18-415-009	KY433693	91.9	2014	Niger	CV-A20
NIG-18-415-010	KX765171	92.6	2015	Nigeria	EV-A119
NIG-18-415-011	MH084295	91.3	2013	Senegal	EV-A120
NIG-18-415-012	MG845889	96.0	2017	USA	CV-B4
NIG-18-415-013	MK836121	92.9	2018	USA	CV-A2
NIG-18-415-014	MN718837	97.9	2019	Nigeria	CV-B2
NIG-18-415-015	JX417870	84.3	2008	Cameroon	CV-A24
NIG-18-415-016	KF303098	86.9	2012	Nigeria	CV-A13
NIG-18-415-017	KX580687	94.3	2014	Nigeria	E-26
NIG-18-415-018	JX476261	84.7	2010	India	E-26
NIG-18-415-019	JX426645	83.5	2008	Cameroon	CV-A13
NIG-18-415-020	KY748288	85.7	2015	Nigeria	CV-A17
NIG-18-415-021	MF925355	93.7	2013	Nigeria	E-12
NIG-18-415-022	KX580662	95.0	2014	Nigeria	E-13
NIG-18-415-023	MN718836	98.2	2019	Nigeria	E-7
NIG-18-415-024	JX417874	83.1	2008	Cameroon	CV-A24
NIG-18-415-025	MN718838	92.6	2019	Nigeria	E-24
NIG-18-415-026	JN203849	89.4	2008	India	E-18
NIG-18-415-027	KX765171	92.9	2015	Nigeria	EV-A119
