# Supplementary material for: High Diversity of Human Non-Polio Enterovirus Serotypes Identified in Contaminated Water in Nigeria
Source: Viruses. 2021 Feb 5;13(2):249. doi: 10.3390/v13020249 (PMC7914538; doi:10.3390/v13020249)
Supplement: Supplementary file 1 [file viruses-13-00249-s001.zip › Table S2.pdf]

Table S2. Distribution of EV serotypes identified in different surveillance studies in Nigeria.

S/N	Serotype	2002-2003 AFC/HC	2010 ES	2010 AFP	2012 ES	2012 AFP	2013 ES	2013 AFP	2014 HC	2014 AFP	2015 AFP	2016 2017 HC	2017 AFP	2017 2018 ES
1	CV-A1								YES		YES			
2	CV-A2													YES
3	CV-A3	YES							YES					
4	CV-A4	YES							YES				YES	YES
5	CV-A5													YES
6	CV-A6	YES											YES	YES
7	CV-A8								YES					
8	CV-A9			YES										
9	CV-A10										YES			
10	CV-A12													YES
11	CV-A14													YES
12	EV-A71	YES							YES	YES				YES
13	EV-A76												YES	YES
14	EV-A119										YES			YES
15	EV-A120									YES				YES
16	CV-B1					YES								
17	CV-B2					YES				YES			YES	YES
18	CV-B3	YES					YES			YES				
19	CV-B4			YES		YES				YES	YES			YES
20	CV-B5	YES			YES					YES				
21	CV-B6	YES		YES	YES									
22	E-1				YES					YES			YES	YES
23	E-2								YES					
24	E-3			YES		YES	YES			YES				YES
25	E-5									YES				
26	E-6	YES	YES		YES	YES	YES	YES		YES	YES			YES
27	E-7		YES	YES	YES	YES	YES	YES		YES	YES			YES
28	E-9												YES	YES
29	E-11	YES	YES	YES	YES	YES	YES			YES				
29	E-12	YES	YES	YES			YES			YES				YES
30	E-13	YES	YES	YES	YES	YES				YES	YES			YES
31	E-14	YES		YES						YES	YES			YES
32	E-15			YES										YES
33	E-17									YES				
34	E-18											YES		YES
35	E-19	YES	YES	YES	YES	YES	YES			YES	YES			YES
36	E-20	YES	YES		YES		YES			YES				
37	E-21	YES								YES	YES			
38	E-23	YES												
39	E-24	YES		YES						YES				YES
40	E-25										YES			YES
41	E-26	YES								YES				YES
42	E-27					YES								
43	E-29	YES		YES		YES					YES			
44	E-30	YES				YES				YES				
45	E-31										YES			YES
46	E-32								YES					
47	E-33	YES			YES	YES				YES				
48	EV-B69	YES												
49	EV-B73									YES				YES
50	EV-B74													YES
51	EV-B75				YES					YES	YES			
52	EV-B80								YES	YES				YES

53	EV-B81													YES
54	EV-B85													YES
55	EV-B86													YES
56	EV-B87													YES
57	EV-B88								YES					
58	EV-B93									YES				
59	EV-B97			YES						YES	YES			YES
60	EV-B106													YES
61	EV-B111										YES			
62	CV-A11										YES			YES
63	CV-A13				YES		YES				YES	YES	YES	YES
64	CV-A17	YES									YES		YES	YES
65	CV-A19										YES			YES
66	CV-A20				YES				YES		YES	YES		YES
67	CV-A24		YES								YES	YES		YES
68	EV-C99								YES	YES	YES	YES		YES
69	EV-C116										YES	YES		YES
70	EV-D94												YES	
71	EV-D111												YES	YES

Colour Code

	First detected in Nigeria in this study
	Detected in AFP 2010/2012 (Unpublished)
	First detected in Nigeria in AFP 2012 (Unpublished)

References

1. Oyero OG, Adu FD, Ayukekbong JA. Molecular characterization of diverse species enterovirus-B types from children with acute flaccid paralysis and asymptomatic children in Nigeria. Virus Res. 2014 Aug 30;189:189-93. doi: 10.1016/j.virusres.2014.05.029. Epub 2014 Jun 7. PMID: 24915283.
2. Adeniji JA, Faleye TO. Isolation and identification of enteroviruses from sewage and sewage-contaminated water in Lagos, Nigeria. Food Environ Virol. 2014 Jun;6(2):75-86. doi: 10.1007/s12560-014-9137-5. Epub 2014 Feb 25. PMID: 24566762.
3. Adeniji JA, Faleye TO. Impact of cell lines included in enterovirus isolation protocol on perception of nonpolio enterovirus species C diversity. J Virol Methods. 2014 Oct;207:238-47. doi: 10.1016/j.jviromet.2014.07.016. Epub 2014 Jul 24. PMID: 25064357.
4. Faleye TO, Adeniji JA. Enterovirus Species B Bias of RD Cell Line and Its Influence on Enterovirus Diversity Landscape. Food Environ Virol. 2015 Dec;7(4):390-402. doi: 10.1007/s12560-015-9215-3. Epub 2015 Sep 24. PMID: 26403309.
5. Faleye TO, Adewumi MO, Coker BA, Nudamajo FY, Adeniji JA. Direct Detection and Identification of Enteroviruses from Faeces of Healthy Nigerian Children Using a Cell-Culture Independent RT-Seminested PCR Assay. Adv Virol. 2016;2016:1412838. doi: 10.1155/2016/1412838. Epub 2016 Mar 20. PMID: 27087810; PMCID: PMC4818813.
6. Adeniji JA, Oragwa AO, George UE, Ibok UI, Faleye TOC, Adewumi MO. Preponderance of enterovirus C in RD-L20B-cell-culture-negative stool samples from children diagnosed with acute flaccid paralysis in Nigeria. Arch Virol. 2017 Oct;162(10):3089-3101. doi: 10.1007/s00705-017-3466-2. Epub 2017 Jul 10. PMID: 28691129.
7. Faleye TOC, Adewumi MO, Japhet MO, David OM, Oluyege AO, Adeniji JA, Famurewa O. Non-polio enteroviruses in faeces of children diagnosed with acute flaccid paralysis in Nigeria. Virol J. 2017 Sep 12;14(1):175. doi: 10.1186/s12985-017-0846-x. PMID: 28899411; PMCID: PMC5596853.

8. Adeniji JA, Ayeni FA, Ibrahim A, Tijani KA, Faleye TOC, Adewumi MO. Comparison of Algorithms for the Detection of Enteroviruses in Stool Specimens from Children Diagnosed with Acute Flaccid Paralysis. J Pathog. 2017;2017:9256056. doi: 10.1155/2017/9256056. Epub 2017 Dec 28. PMID: 29445548; PMCID: PMC5763071
9. Donbraye E, Olasunkanmi OI, Opabode BA, Ishola TR, Faleye TOC, Adewumi OM, Adeniji JA. Abundance of enterovirus C in RD-L20B cell culture-negative stool samples from acute flaccid paralysis cases in Nigeria is geographically defined. J Med Microbiol. 2018 Jun;67(6):854-865. doi: 10.1099/jmm.0.000737. Epub 2018 Apr 30. PMID: 29708482.
10. Adewumi OM, Faleye TOC, Okeowo CO, Oladapo AM, Oyathelemhi J, Olaniyi OA, Isola OC, Adeniji JA. Identification of previously untypable RD cell line isolates and detection of EV-A71 genotype C1 in a child with AFP in Nigeria. Pathog Glob Health. 2018 Dec;112(8):421-427. doi: 10.1080/20477724.2018.1548117. Epub 2018 Nov 26. PMID: 30474520; PMCID: PMC6327568.
11. Osundare FA, Opaleye OO, Akindele AA, Adedokun SA, Akanbi OA, Bock CT, Diedrich S, Böttcher S. Detection and Characterization of Human Enteroviruses, Human Cosaviruses, and a New Human Parechovirus Type in Healthy Individuals in Osun State, Nigeria, 2016/2017. Viruses. 2019 Nov 7;11(11):1037. doi: 10.3390/v11111037. PMID: 31703317; PMCID: PMC6893832.
12. Adewumi M.O., Ogunsakin T.R., Ogunrombi S.B., Ojeamiren I., Olawole S.A., Faleye T.O.C., Adeniji J.A. 2019. Detection of Non-cytopathic enteroviruses in supernatant of RD and L20B cell cultures. Hosts and Viruses, 6(4): 93-99.
13. Faleye,T.O.C., Adewumi, M. O., Olayinka.O. T. and Adeniji, J. A. 2019. Isolation and complete capsid sequence of enterovirus D111 from faeces of a child with acute flaccid paralysis in Nigeria. Hosts and Viruses, 6(4): 85-92.
