# Supplementary material for: High Diversity of Human Non-Polio Enterovirus Serotypes Identified in Contaminated Water in Nigeria
Source: Viruses. 2021 Feb 5;13(2):249. doi: 10.3390/v13020249 (PMC7914538; doi:10.3390/v13020249)
Supplement: Supplementary file 1 [file Figure S1.pdf › Figure S1.pdf]

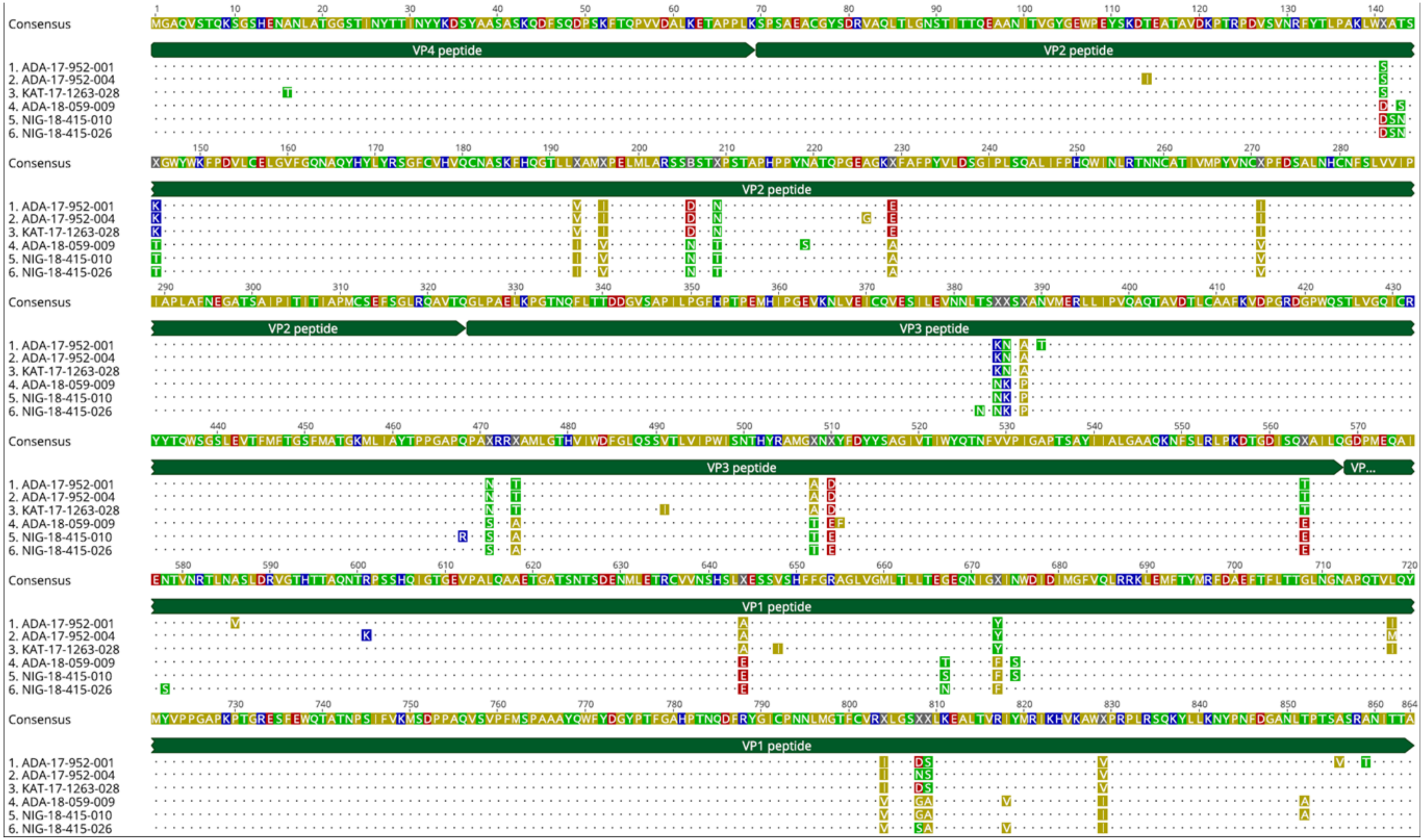

Supplementary Figure 1. Alignment of capsid amino acid sequences from 6 EV-119 strains from Nigerian sewage samples. Differences from consensus sequences are shown. The location of VP1-VP4 peptides is indicated in green boxes.
